# Absence of cyclin-dependent kinase inhibitor *p27* or *p18* increases efficiency of iPSC generation without induction of iPSC genomic instability

**DOI:** 10.1038/s41419-019-1502-8

**Published:** 2019-03-20

**Authors:** Zhiyan Zhan, Lili Song, Weiwei Zhang, Haihui Gu, Haizi Cheng, Yingwen Zhang, Yi Yang, Guangzhen Ji, Haizhong Feng, Tao Cheng, Yanxin Li

**Affiliations:** 1 Key Laboratory of Pediatric Hematology and Oncology Ministry of Health, Department of Hematology & Oncology, Pediatric Translational Medicine Institute, Shanghai Children’s Medical Center, School of Medicine, Shanghai Jiao Tong University, Shanghai, 200127 China; 2 State Key Laboratory of Oncogenes and Related Genes, Renji-Med X Clinical Stem Cell Research Center, Ren Ji Hospital, School of Medicine, Shanghai Jiao Tong University, Shanghai, 200127 China; 3 Department of Blood Transfusion, Changhai Hospital, Shanghai, 200433 China; 4 Department of Radiation Oncology, University of Pittsburgh School of Medicine, University of Pittsburgh Cancer Institute, 5117 Center Avenue, Pittsburgh, PA 15213 USA; 5 State Key Laboratory of Experimental Hematology, Institute of Hematology and Blood Diseases Hospital, Chinese Academy of Medical Sciences and Peking Union Medical College, Tianjin, China; 6 Center for Stem Cell Medicine, Chinese Academy of Medical Sciences, Tianjin, China; 7 Department of Stem Cell & Regenerative Medicine, Peking Union Medical College, Tianjin, China

## Abstract

Mechanisms underlying the generation of induced pluripotent stem cells (iPSC) and keeping iPSC stability remain to be further defined. Accumulated evidences showed that iPSC reprogramming may be controlled by the cell-division-rate-dependent model. Here we reported effects of absence of mouse *p27* or *p18* on iPSC generation efficiency and genomic stability. Expression levels of cyclin-dependent kinases inhibitors (CDKIs), p21, p27, and p18 decreased during iPSC reprogramming. Like *p21* loss, *p27* or *p18* deficiency significantly promoted efficiency of iPSC generation, whereas ectopic expression of p27, p18, or treatment with CDK2 or CDK4 inhibitors repressed the reprogramming rate, suggesting that CDKIs-regulated iPSC reprogramming is directly related with their functions as CDK inhibitors. However, unlike *p21* deletion, absence of *p27* or *p18* did not increase DNA damage or chromosomal aberrations during iPSC reprogramming and at iPSC stage. Our data not only support that cell cycle regulation is critical for iPSC reprogramming, but also reveal the distinction of CDKIs in somatic cell reprogramming.

## Introduction

The reprogramming of somatic cells into induced pluripotent stem cells (iPSC) by introduction of the four defined transcription factors (Oct4, Sox2, Klf4, and c-Myc) is an intensively investigated area in stem cell research for its enormous potential in regenerative medicine since 2006^1^. The efficiency of iPSC generation however remains low, and the iPSC genomic stability is still concerned.

Accumulated evidences demonstrated that iPSC reprogramming is mainly controlled by the cell-division-rate-dependent model^2–7^. p21, p27, and p18 are three important cell cycle regulators and cyclin-dependent kinase inhibitors (CDKIs)^8–12^. Limited studies showed that p21, p27, and p18 are important for iPSC reprogramming^2,13–16^. We and others demonstrated previously that loss of *p21* could promote somatic reprogramming, however, caused markedly genomic instability^2,13,14^. Deletion of *p27* enhances somatic reprogramming in the absence of ectopic Sox2^15^. p18 reduces iPSC reprogramming by targeting CDK4/6-mediated cell cycle regulation^16^. However, their roles in controlling iPSC quality and genomic stability are still unclear.

Here we examined iPSC generation from murine cells that are deficient in *p27* or *p18* in comparison with *p21* loss. We found that although loss of different CDKIs can improve iPSC colony formation efficiency, but iPSC quality with loss of *p21*, *p27*, or *p18* was significantly different. In comparison to loss of *p21*, iPSC with absence of *p27* or *p18* were associated with fewer chromosomal aberrations. Our results demonstrated that deletion of *p27* or *p18* may be a better choice to enhance iPSC generation efficiency with a guaranteed quality.

## Methods

### Mice

Wild type, *p21*^−/−^, *p18*^−/−^, *p27*^−/−^, and NOD/SCID/Gamma (NSG) mice were purchased from The Jackson Laboratory. All the mice strains had been crossed to generate a pure C57BL/6J background and maintained in the pathogen free animal facility, Institute of Hematology and Blood Diseases Hospital. All procedures and animal experiments were approved by the Institutional Animal Care and Use Committee at Institute of Hematology and Blood Diseases Hospital, CAMS/PUMC.

### Cells culture conditions

Primary MEFs were obtained from 13.5-day embryos of the indicated genotypes based on the protocol from Wicell and cultured in standard DMEM medium containing 10% FBS (Millipore) in our lab. Murine ES cell was purchased from Wicell and iPS cells were reprogramming from MEF cells with four factors (Oct4, Sox2, Klf4, and c-Myc). Murine ES and iPSC cells were cultured in ‘ES culture medium’ composed of Knock-Out DMEM (Invitrogen) supplemented with ES cell qualified FBS (20%, Millipore), mouse LIF (1000 U/ml), non-essential amino acids, L-Glutamine, and β-mercaptoethanol. Bone marrow c-kit^+^ cells were harvested from femurs of mice, enriched by CD177-streptavidin kit (Miltenyi), and cultured in standard BIT9500 (Stemcell Technologies) containing 10% FBS supplemented with murine SCF, Flt-3, and TPO before transduction.

### Plasmids

Retroviral constructs pMXs-Klf4 (#13370)^1^, pMXs-Sox2 (#13367)^1^, pMXs-Oct4 (#13366)^1^, pMXs-c-Myc (#13375)^1^, were obtained from Addgene. The cDNA of mouse *p27* and *p18* were also purchased from Addgene. They were both cloned into pMXs-GFP retroviral vectors.

### Generation of mouse iPS cells

Reprogramming of primary (passage 2) MEFs was performed as previously described^1^. In brief, primary MEFs of indicated genotypes were seeded in 100-mm-diameter dish (5 × 10^5^ cells per dish) pre-coated with 0.1% gelatin (Sigma). They were transduced twice in the next two days at 24-h interval by virus supernatant collected from Plat-E cells transfected with the previously mentioned retroviral plasmids. At the end of transduction, medium was changed to ES culture medium. After cultured for 10–12 days, colonies with ES-cell-like morphology became visible. They were then either scored after SSEA1 staining or picked for further expansion on feeder fibroblasts using standard ES culture methods.

### Reprogramming efficiency analysis

For quantification of iPSC generation efficiency, retroviral transduction was measured in parallel infections containing all the retroviruses used for reprogramming plus a GFP retrovirus (pMXs-GFP) (equal volumes of each retrovirus were used during the transduction). The efficiency of transduction was measured by FACS analysis the next day after medium was changed to ES culture medium. Total numbers of iPSC colonies were counted after staining plates for SSEA1 antibody (R&D). Briefly, 5 × 10^5^ cells per 100-mm dish were seeded after retroviral transduction and measured GFP positive rates in different genotypes. The numbers of SSEA1^+^ colonies were counted on Day 12. The percentage of SSEA1^+^ colonies over all the transduced MEFs was determined. The efficiency of reprogramming was also calculated as the relative change compared to that of control.

### CDK inhibitors and p27 siRNA sequence

CDK4 (CDK4/6) inhibitor Palbociclib (PD-0332991, Cat#: S1116, Selleck), CDK2 (Cdc2, CDK2, and CDK5) inhibitor Roscovitine (Seliciclib, CYC202, Cat: S1153, Selleck), CDK4/6 inhibitor Abemaciclib (Cat: HY-16297, MCE), CDK7 inhibitor THZ1 (Cat: S7549, Selleck), p18 inhibitor NSC23005 sodium (Cat: HY-100791, MCE). p27 siRNA sequence: 5′-GTGGAATTTCGACTTTCAG-3′.

### Teratoma formation

Cells (2 × 10^6^) of indicated mouse iPS cell lines were subcutaneously injected into NOD/SCID mice. Teratomas were recovered and surgically removed after 3 weeks. Tissues were snap-frozen, embedded in tissue-tek with O.C.T. compound, and stored at −80 °C. The samples were sectioned at a thickness of 5 mm and stained with haematoxylin and eosin for pathological examination.

### Western blot analysis

Cell extracts were prepared using RIPA buffer, resolved on NuPAGE 12% gradient Bis-Tris gels, transferred to nitrocellulose and hybridized using antibodies against p27 (1:500), p18 (1:500), p21 (1:500), RAD51 (1:500), and β-actin (1:500 dilution, Santa Cruz); PARP (1:1000 dilution, cell signaling technology); γH2AX-S139 (1:1000) and, 53BP1 (1:1000, abcam).

### Immunofluorescence

Cells of mouse iPS cell lines were cultured in 12-well plates with feeders for 2–3 days until colonies formed. The cells were then fixed in 4% paraformaldehyde for 15 min, permeabilized with 0.3% Triton X-100 in PBS for 15 min and blocked with 5%BSA in PBS for 1 h at room temperature. After incubation with antibodies against mouse Oct-4, Sox-2, Nanog (1:100 diluted in PBS containing 4%BSA, Santa Cruz) overnight at 4 °C, cells were washed with PBS the next day and incubated with secondary antibodies conjugated with Alexa 488 or Alexa 555 (1:1000 diluted in PBS containing 4% BSA, Molecular Probes). For staining of SSEA-1(R&D), the permeabilization step was emitted.

### Detection of double-strand breaks of DNA

Reprogramming of MEFs of indicated genotypes with the four factors was performed as we previously described. Reprogramming cells were digested by trypsin and resuspended in PBS. SSEA1^+^ cells were sorted and then cytospun onto the slides. Or four genotype iPS cell lines were irradiated with 2 Gy and collected at indicated time points. The slides were then fixed in 4% paraformaldehyde for 15 min, permeabilized by 0.3% Triton X-100 in PBS, and blocked by 5% BSA in PBS for 1 h at room temperature. The slides were incubated with mouse monoclonal antibodies against γH2AX (1:150 diluted in PBS containing 2% BSA, Trevigen) overnight at 4 °C and secondary antibodies conjugated with Alex-555 for 1 h at room temperature the next day. DAPI was used as nuclear staining. Images were produced from confocal microscope (Leica) at ×63 magnification and analyzed for foci/nucleus.

### Comet assay

At reprogramming Day 12, the cells were collected and digested. Then sorted SSEA1^+^ positive cells were sorted. Or four genotype iPS cell lines were irradiated with 2 Gy, collected at different time points. The cells were then processed for alkaline comet assay using Comet SCGE Assay kit (Enzo Life Sciences) according to the manufacturer’s protocol. Each slide was photographed under a Zeiss Axio Observer Z1 microscope and the percentage of tail intensity was computed by the Comet Assay IV software (Perceptive Instruments Ltd.).

### Quantitative real-time PCR analysis

For the determination of mRNA levels of p18, p21, and p27 during reprogramming and in MEF, ESC, and iPS cells, cells were harvested by treating with trypsin-EDTA solution and washed with PBS three times, SSEA1^+^ cells were sorted at the different time points during reprogramming. Total RNA was extracted by using RNeasy kit (Qiagen) according to the manufacturer’s instructions. RNA was treated with RNase–free DNase (Invitrogen) for 15 min at room temperature before reverse transcription with superscript II RT (Invitrogen). Real-time PCR was performed on the chromo 4^TM^ detector (M J Research) with SYBR Green PCR master mix (Thermo Scientific). PCR conditions consisted of a 10-min hot start at 95 °C followed by 40 cycles of 95 °C for 15 s, 60 °C for 1 min and incubation for 3 s at 77 °C with a final extension for 10 min at 72 °C. The average threshold cycle (Ct) for each gene was determined from triplicate reactions and the levels of gene expression relative to β-actin were determined as we previously described^17^. *p18* primers: 5′-CTCCGGATTTCCAAGTTCA-3′ and 5′-GGGGGACCTAGAGCAACTTAC-3′. *p21* primers: 5′-GTGGGTCTGACTCCAGCCC-3′ and 5′-CCTTCTCGTGAGAC GCTTAC-3′. *p27* primers: 5′-CGATCGGAATTCATGTCAAACGTGCGAGTG-3′ and 5′-CGATCGAGATCTTTACGTTTGACGTCTTCTGAGGCC-3′. *ACTB* primers: 5′-ATGGAGGGGAATACAGCCC-3′ and 5′-TTCTTTGCAGCTCCTTCGTT-3′.

### Cell cycle and proliferation analysis

Cell cycle and proliferation analysis assays were performed using Click-iT EdU Assay Kits at the indicated time points during reprogramming as we previously described^13^. We selected pacific blue to show the EDU and 7-AAD to stain the DNA. The flow cytometry data were analyzed by Syan software.

### Karyotyping and G-banding assays

G-banding chromosome analysis of the iPS cell lines were performed as we previously described^13^. Data were interpreted by a certified cytogenetic technologist.

### Centrosome number and spindle assays

Cells growing on coverslips were irradiated at the different time point. The cells were fixed with prechilled methanol for 20 min at −20 °C, washed with phosphate buffered saline (PBS), and permeabilized with 1% NP-40 in PBS for 5 min at 25 °C. Cells were blocked with 10% normal goat serum in PBS for 1 h and probed with anti-γ-tubulin monoclonal antibody (1:400, GTU-88, Sigma) for 1 h at 25 °C. The antibody–antigen complexes were detected with secondary antibodies conjugated with Alexa 555 (1:1000 diluted in PBS containing 4% BSA, Molecular Probes) by incubation for 1 h at 25 °C. The samples were counterstained with DAPI. These panels show merge of DNA (blue) and γ-tubulin (red). Scale bars, 10 mm. for showing the spindle, based on centrosome staining, added α-Tubulin antibody (1:400, Sigma) conjugated with FITC to show the spindle fibers (green).

### Statistical analyses

Data are expressed as mean ± SD. All analyses were two-tailed and considered statistically significant when *P* values were less than or equal to 0.05.

## Results

### Expression levels of p21, p27, and p18 are decreased during iPSC reprogramming

**Fig. 1:**
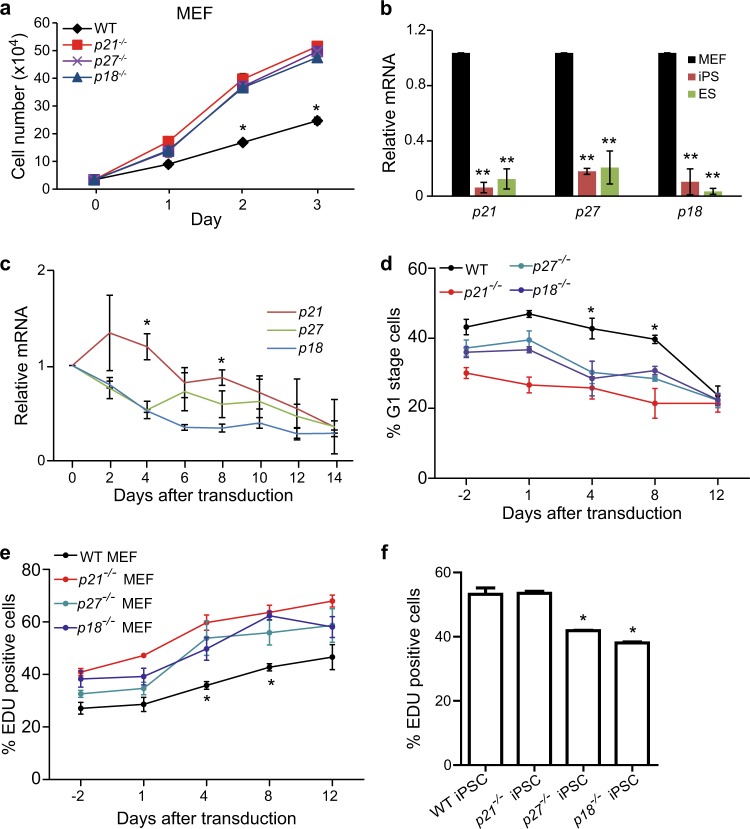
Expression levels of *p21*, *p27*, and *p18* are decreased during reprogramming. **a** Cell proliferation analysis of MEFs wild type (WT), *p21*^−/−^, *p27*^−/−^, or *p18*^−/−^ mutant. Cell proliferation was determined by counting cell numbers using trypan blue staining. **b** qRT-PCR analysis of the mRNA expression levels of *p18*, *p27*, and *p21* in mouse WT ESCs, iPSCs and MEFs. **c** Expression levels of *p18*, *p27*, and *p21* mRNA in WT MEFs at indicated time points during reprogramming. **d** Flow cytometry (FACS) analysis of cell cycle using EDU and 7-AAD staining at the indicated time points during reprogramming. **e** Proliferation measured by EDU staining in different genotype MEFs at different time points during reprogramming. **f** Proliferation measured by EDU staining in different genotype iPSCs. Data are representative of two or three independent experiments. Error bars, ±SD. *p < 0.05; **p < 0.01, by two-tailed *t* test

To determine the roles of CDKIs, p21, p27, and p18 in iPSC reprogramming, we first tested cell proliferation in wild type (WT), *p21*^−/−^, *p27*^−/−^, and *p18*^−/−^ MEFs. As shown in Fig. 1a, compared to WT, deletion of CDKIs all promoted cell proliferation. Then, we assessed the mRNA expression of *p21*, *p27*, and *p18* in MEF, iPSC, and ESC derived from the same mice, and found that all CDKIs expressions were lower in iPSC and ESC compared to MEFs (Fig. 1b). We further tested the mRNA expression of *p21*, *p27*, and *p18* during iPSC reprogramming, and found that all CDKIs expression levels were reduced (Fig. 1c). Moreover, at the early stage (Day 4) and middle stage (Day 8) after transduction, *p21* expression was relatively higher than *p27* and *p18* (Fig. 1c). This suggests that these CDKIs’ expression levels are negatively correlated with cell stemness and pluripotency.

Next, we examined the effects of deletion of CDKIs on cell cycle progression during iPSC reprogramming. As shown in Fig. 1d, e, the percentages of G1-phase cells of all genotypes were decreased and the cell proliferation rates were increased. However, at the early stage (Day 4) and middle stage (Day 8) after transduction, the percentages of G1 stage cells in the group with *p21*^−/−^, *p27*^−/−^, and *p18*^−/−^ genotypes were markedly lower than that with WT genotype (Fig. 1d), which was correlated with increased cell proliferation rates measured by EDU incorporation (Fig. 1e). We further determine effects of *p21*^−/−^, *p27*^−/−^, or *p18*^−/−^ KO on iPSC proliferation. As shown in Fig. 1f, *p21* KO did not affect iPSC proliferation and *p18* or *p27* KO significantly inhibited iPSC proliferation compared to WT. These data show that expression levels of CDKIs are decreased during iPSC reprogramming and thereby cell cycle and proliferation of reprogramming cells with absence of CDKIs are changed.

### Deletion of CDKIs increases iPSC reprogramming efficiency

**Fig. 2:**
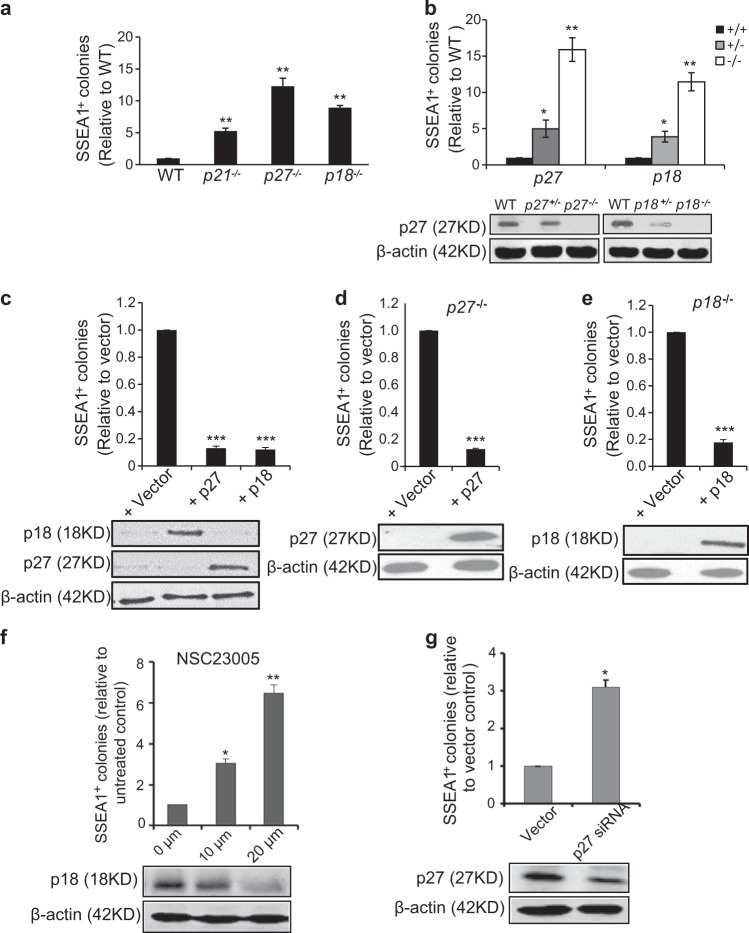
Absence of CDKIs increases iPSC reprogramming efficiency. **a** SSEA1 positive colonies in *p21*^−/−^, *p27*^−/−^, and *p18*^−/−^ iPSCs relative to that of WT iPSCs at day 14 after transduction with four factors. **b** Upper, SSEA1 positive colonies in *p27*^+/−^, *p27*^−/−^, *p18*^+/−^, and *p18*^−/−^ iPSCs relative to that of WT iPSCs at day 14 after transduction with four factors. Lower, Western blot analysis of endogenous expression of p27 and p18 in various genotype MEFs. **c**–**e** Effects of ectopic expression of p27 or p18 on iPSC generation in WT, *p18*^−/−^, and *p27*^−/−^ MEFs. Upper, numbers of SSEA1 positive colonies. Lower, Western blots analysis of ectopic expression of p18 and p27 in various genotype MEFs at day 4 of reprogramming after transduced with 4F and p27 or p18. **f** Effects of p18 inhibitor on iPSC generation in WT MEFs. Upper, numbers of SSEA1 positive colonies relative to untreated control. Lower, Western blots analysis of p18 expression under p18 inhibitor treated at day 4 of reprogramming after transduced with 4F. **g** Effects of p27 siRNA on iPSC generation in WT MEFs. Upper, numbers of SSEA1 positive colonies relative to vector control. Lower, Western blots analysis of p27 expression at day 4 of reprogramming after transduced with 4F and p27 SiRNA. Data are representative of two or three independent experiments. Error bars, ±SD. *p < 0.05; **p < 0.01, by two-tailed *t* test

To compare iPSC reprogramming efficiency of MEFs with *p21*^−/−^, *p27*^−/−^, and *p18*^−/−^ genotypes, we measured the quantities of SSEA1^+^ iPSC colonies. As shown in Fig. 2a, like *p21* loss, absence of *p27* or *p18* increased iPSC reprogramming efficiency compared to WT. SSEA1^+^ iPSC colonies with *p27*^−/−^ or *p18*^−/−^ all expressed pluripotency markers, Oct4, Nanog and Sox2, (Supplementary Figure 1a) and produced teratomas that could differentiate into all three germ layers (Supplementary Figure 1b). Moreover, absence of either *p27* or *p18* increased iPSC generation to an extent comparable to that of *p21* deficiency not only with four factors (Oct4, Sox2, Klf4, and c-Myc) but also three factors (Oct4, Sox2, and Klf4) (Fig. 2a and Supplementary Figure 2a). Besides, *p27* or *p18* deletion mediated increase in iPSC generation efficiency was dose dependent (Fig. 2b and Supplementary Figure 2b). Ectopic expression of p27 or p18 in WT, *p27*^−/−^or *p18*^−/−^ genotype MEFs all reduced iPSC reprogramming efficiency (Fig. 2c–e). To further validate these observations, we used p18 small-molecular inhibitor NSC23005 and p27 siRNA to treat during the reprogramming of WT MEFs. As shown in Fig. 2f, g, both NSC23005 treatment and p27 siRNA knockdown significantly promoted iPSC generation. These data showed that all CDKIs are critical for iPSC reprogramming, and reprogramming efficiency has no difference among them.

### Small-molecule inhibitors of CDKs can reduce iPSC generation as CDKIs

**Fig. 3:**
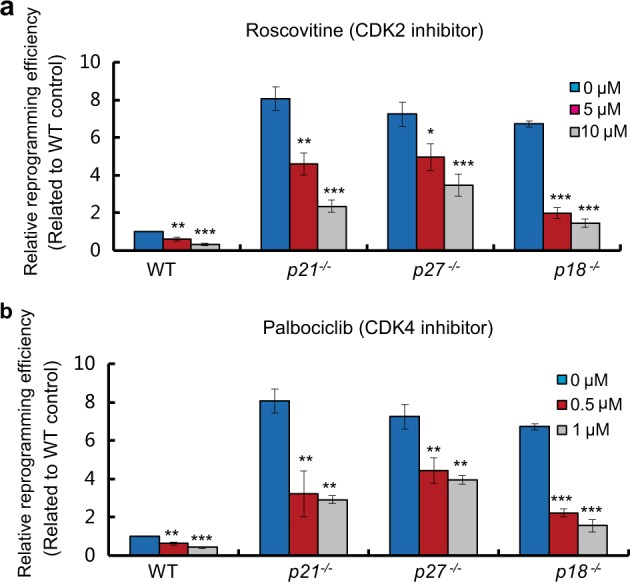
Small-molecule inhibitors of CDKs reduce iPSC generation as CDKIs. **a**, **b** Effects of CDK2 (**a**) and CDK4 (**b**) inhibitor treatment on efficiency of iPSC generation in WT, *p21*^−/−^, *p27*^−/−^, and *p18*^−/−^ MEFs. Inhibitors were added in the medium during reprogramming. Data are representative of two or three independent experiments. Error bars, ± SD. *p < 0.05; **p < 0.01, ***p < 0.001 by two-tailed *t* test

p21 and p27 are inhibitors of broad cyclin-CDK complexes, and p18 is a specific inhibitor of CDK4/6 kinases^8,11,18,19^. To test whether roles of p21, p27, and p18 in iPSC reprogramming are directly related with them as CDK inhibitors, we used small-molecule CDK2 and CDK4 inhibitors to treat MEFs with various genotypes during reprogramming. As shown in Fig. 3a, b, both the treatment of Small-molecule CDK2 and CDK4 inhibitors reduced all genotypes iPSC reprogramming efficiency. We also found that treatment with both CDK4 and CDK2 inhibitors almost blocked iPS generation in WT MEFs (supplemental Fig. 3a). To further validate CDK inhibitors’ function in iPS generation, the small-molecule CDK4/6 (Abemaciclib) and CDK7 inhibitors (THZ1) were used to treat WT MEFs with CDK4/6 small-molecule inhibitor Abemaciclib or CDK7 small-molecule inhibitor THZ1, respectively. As shown in supplemental Fig. 3b and c, both Abemaciclib and THZ1 reduced iPSC reprogramming efficiency. These results suggest that CDKIs-regulated iPSC reprogramming is directly related with their functions as CDK inhibitors.

### p27 and p18 are dispensable for genomic stability during reprogramming

**Fig. 4:**
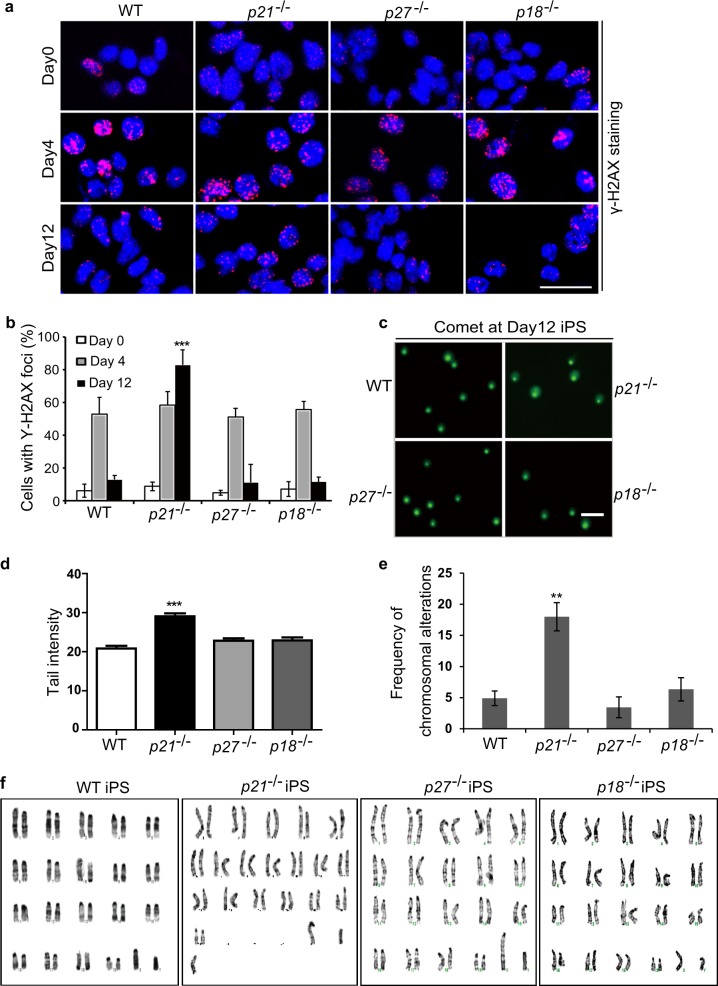
p27 and p18 are dispensable for genomic stability during reprogramming. **a** Representative images of γ-H2AX staining in various genotypes cells at reprogramming Day 0, Day 4, and Day 12. Bars: 50 μm. **b** Percentage of cells with γ-H2AX foci in (**a**). **c** Representative images of comet assay in various genotype cells at reprogramming Day 12. Bars: 200 μm. **d** Tail intensity of the cells in (**c**). **e** The karyotypic alterations in three clones each from three independent iPSCs at passage 5 derived from three passage 3 MEF cell lines per genotype were quantified. Graphic illustration of the number of chromosomal alterations (numerical gains, losses, and structural alterations) in the iPSCs. **f** Karyotypes from 20 metaphase cells from three sets of each three cell lines per type of iPSCs were analyzed under the microscope or after digital imaging and karyotyping. Images shown were taken with ×100 oil objective. Data are representative of two or three independent experiments. Error bars, ±SD. **p < 0.01, ***p < 0.001, by two-tailed *t* test

DNA damage frequently occurs during somatic reprogramming^5,20–24^. We and others previously showed that absence of p53 or p21 could not only improve the reprogramming efficiency but also increase genomic instability^13,14^. p27 and p18 were also shown to be related with DNA damage^25–29^. To compare DNA damage status in various genotype MEFs during reprogramming, we examined the DNA double-strand breaks using anti-γ-H2AX staining in the transduced MEFs at Day 0, Day 4 and the iPSC colonies at Day 12 after transduction (Fig. 4a, b). There was no difference among all genotype cells at reprogramming Day 0 and Day 4, whereas γ-H2AX foci formation in *p21*^−/−^ iPSC colonies at reprogramming Day 12 significantly increased when compared with other genotypes (Fig. 4a, b). The results at reprogramming Day 12 were validated using comet assays (Fig. 4c, d). These data suggest that unlike p21, p27, and p18 are dispensable for DNA damage repair during iPSC reprogramming.

We further quantified the chromosomal alterations in three clones, each from three independent iPSCs at passage 5 derived from three passage 3 MEF cell lines per genotype. Chromosomal alterations, including chromosomal gains, losses, and structural abnormalities were observed and quantified in the iPSCs of all genotypes and the corresponding MEFs (Fig. 4e, f, and Supplementary Tables S1 to S6) as we previously described^13^. There was no difference in genomic stability among *p18*^−/−^, *p27*^−/−^, and WT genotypes, and only *p21*^−/−^ caused significantly more chromosomal alterations in comparison to the others. These data showed that p27 and p18 were critical for somatic cell reprogramming efficiency but dispensable for genomic stability during iPSC reprogramming.

### p27 and p18 are dispensable for genomic stability at iPSC stage

**Fig. 5:**
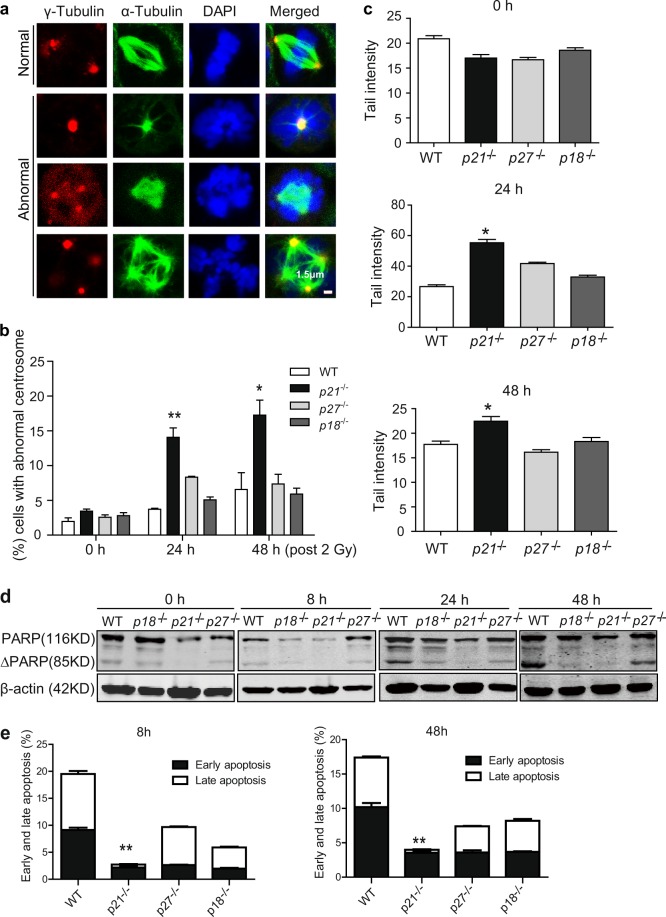
p27 and p18 are dispensable for genomic stability at iPSC stage. **a** Representative images of spindle abnormal phenotype. Bars: 1.5 μm. **b** Abnormal centrosomes in various iPSCs at the indicated time points post 2-Gy IR. **c** Tail intensity of the cells in various iPSCs at the indicated time points post 2-Gy IR. **d** Western blot assays of expression of cleaved PARP in various iPSCs at the indicated time points post 2-Gy IR. **e** FACS analysis of apoptosis at the indicated time points post 2-Gy IR. Data are representative of two or three independent experiments. Error bars, ±SD. **p < 0.01, ***p < 0.001, by two-tailed *t* test

As p27 and p18 do not impair genomic stability during reprogramming, we further determined whether p27 and p18 are also dispensable for genomic stability at iPSC stage. To test this, we selected the best genomic stability cell lines in each genotype and then tested the centrosome number and the spindles after irradiation (IR) treatment. Significantly more abnormal centrosomes and spindles were found only in *p21*^−/−^ iPS cell lines compared with WT, but there was no difference in *p27*^−/−^, *p18*^−/−^, and WT genotype iPS cell lines (Fig. 5a, b).

To confirm the observation, we further tested the percentage of tail intensity in different genotype iPS cell lines after IR treatment. As shown in Fig. 5c–e, loss of *p21* induced significantly higher percentage of tail intensity, less cleaved PARP formation, and less late apoptosis compared to WT, whereas loss of *p18* or *p27* had no effects. These data suggested that p27 and p18 are different from p21 and are dispensable for iPSC genomic stability during reprogramming and at iPSC stage.

## Discussion

Overlapping mechanisms between reprogramming and tumorigenesis which were shown in the p53-p21 pathway represent a huge challenge for the therapeutic use of iPS technology^2,13,14^. The goal of the present study is to gain a better understanding of the roles of CDKIs in iPSC reprogramming and iPSC genomic stability to identify a more suitable molecular target and potentially improve the technology for iPSC generation. We found that compared with loss of *p21*, absence of *p27* or *p18* can promote the efficiency of iPSC generation without the induction of genomic instability.

Successful generation of iPS cells from somatic cells need reset of the patterns of cell cycle in those reprogramming cells, suggesting iPSC reprogramming was mainly controlled by a cell-division-rate-dependent model^6,30^. For example, p53 and its up and downstream regulators such as p16/p19, p21 limit iPSC generation mainly by cell cycle arrest and senescence^2–4^. Here, we found that like p53-p21, absence of *p27* and *p18* promoted efficiency of iPSC generation by regulating cell cycle. Moreover, using small-molecule CDK inhibitor treatment, we revealed that p21, p27, and p18-mediated iPSC reprogramming are directly associated with them as CDKIs.

Although it is known that loss of CDKIs promotes efficiency of iPSC generation, iPSC quality with absence of CDKI’s genotypes is still unclear. p21 was reported to be directly involved in cellular repair processes by binding to proliferating cell nuclear antigen (PCNA) and inhibiting DNA replication after DNA damage^31,32^, and p21 also played a role in aneuploidy formation^33^. *p21*-deficient mice developed spontaneous tumors at an average age of 16 months, whereas wild-type mice were tumor-free beyond 2 years of age^34^. Reduced p27 enhanced chromosomal instability in non-small-cell lung carcinomas and other cancers, and ectopic expression of p27 induces a significantly decrease in the accumulation of aneuploidy^35,36^. The cell cycle inhibitors p21 (Waf1/Cip1) and p27 (Kip1) were frequently downregulated in many human cancers, and correlated with a worse prognosis. Combined deficiency of p21 and p27 proteins in mice was linked to more aggressive spontaneous tumorigenesis, and resulted in a decreased lifespan^37,38^. p18 was also a known haploinsufficient tumor suppressor^39,40^, and loss of *p18* resulted in widespread hyperplasia and organomegaly after birth of the mice^39^. We previously showed that loss of *p21* during reprogramming can induce DNA damage accumulation^13^. Consistent with this, here we demonstrated that loss of *p21* induces iPSC centrosome abnormal and reduces DNA repair ability. However, absence of *p27* or *p18* kept not only mitosis but also chromosome well during reprogramming and at iPSC stage, indicating that p27 and p18 are dispensable for iPSC genomic stability.

In summary, here we have reported a distinct role of CDKIs in somatic cell reprogramming. Deletion of *p27* or *p18* promotes reprogramming efficiency without reduction of genomic stability. Therefore, our results not only support that cell cycle regulation is critical for iPSC reprogramming, but also offer a strong rationale for targeting p27 and p18 in regenerative medicine.

## Supplementary information

Supplemental Tables 1-6

Supplemental Figure 1

Supplemental Figure 2

Supplemental Figure 3
