# Correction: Absence of cyclin-dependent kinase inhibitor *p27* or *p18* increases efficiency of iPSC generation without induction of iPSC genomic instability

**DOI:** 10.1038/s41419-025-07728-4

**Published:** 2025-05-26

**Authors:** Zhiyan Zhan, Lili Song, Weiwei Zhang, Haihui Gu, Haizi Cheng, Yingwen Zhang, Yi Yang, Guangzhen Ji, Haizhong Feng, Tao Cheng, Yanxin Li

**Affiliations:** 1 Key Laboratory of Pediatric Hematology and Oncology Ministry of Health, Department of Hematology & Oncology, Pediatric Translational Medicine Institute, Shanghai Children’s Medical Center, School of Medicine, Shanghai Jiao Tong University, Shanghai, 200127 China; 2 State Key Laboratory of Oncogenes and Related Genes, Renji-Med X Clinical Stem Cell Research Center, Ren Ji Hospital, School of Medicine, Shanghai Jiao Tong University, Shanghai, 200127 China; 3 Department of Blood Transfusion, Changhai Hospital, Shanghai, 200433 China; 4 Department of Radiation Oncology, University of Pittsburgh School of Medicine, University of Pittsburgh Cancer Institute, 5117 Center Avenue, Pittsburgh, PA 15213 USA; 5 State Key Laboratory of Experimental Hematology, Institute of Hematology and Blood Diseases Hospital, Chinese Academy of Medical Sciences and Peking Union Medical College, Tianjin, China; 6 Center for Stem Cell Medicine, Chinese Academy of Medical Sciences, Tianjin, China; 7 Department of Stem Cell & Regenerative Medicine, Peking Union Medical College, Tianjin, China

Correction to: *Cell Death and Disease* 10.1038/s41419-019-1502-8, published online 20 March 2019

In the originally published version of article, the representative the WB images of p18, p27 in Fig. 2C presented the wrong gels. The correct Fig. 2C is enclosed.

**Incorrect Figure 2** 
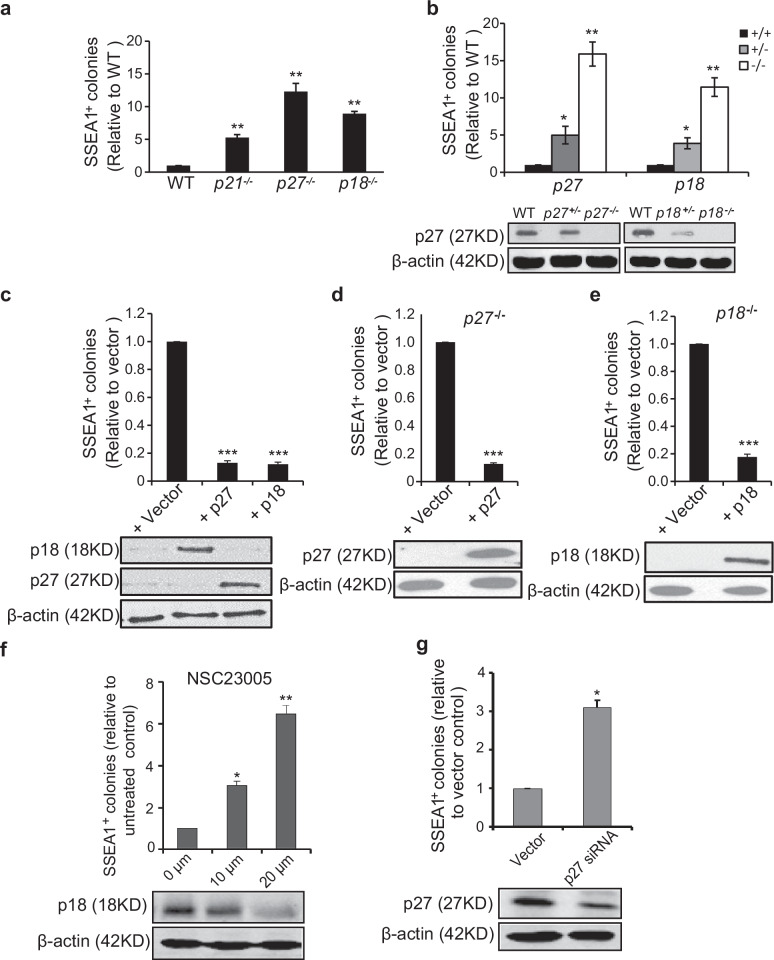


**Correct Figure 2** 
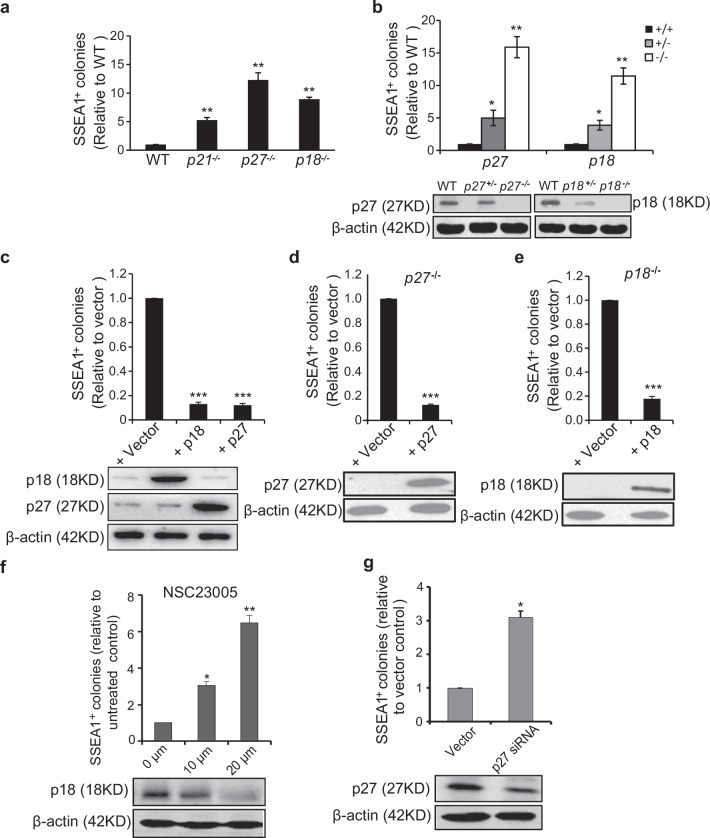


The authors declare that these corrections do not affect the description, interpretation, or the original conclusions of the manuscript.

The authors regret the inconvenience this error may have caused.

