# Supplementary material for: Absence of cyclin-dependent kinase inhibitor p27 or p18 increases efficiency of iPSC generation without induction of iPSC genomic instability
Source: Cell Death Dis. 2019 Mar 20;10(4):271. doi: 10.1038/s41419-019-1502-8 (PMC6426969; doi:10.1038/s41419-019-1502-8)

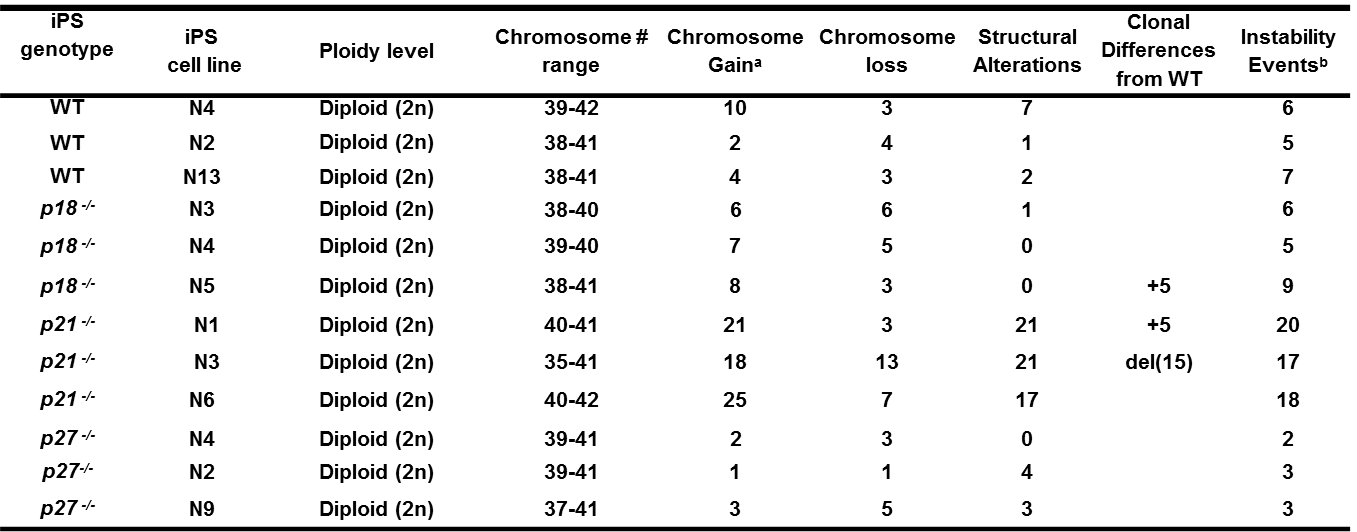
**Supplementary Table S1** Chromosome instability in the four iPSC genotypes (Set #1)

a, The number of chromosomal gains in the 20 cells analyzed. Likewise, loss and structural alterations are represented in the same manner. Figure 4d and associated statistical analyses of chromosomal instability are based on the sum of chromosome gain, loss and structural alterations in each cell lines.

b, Instability events are defined as the number of different cellular events necessary to induce the numerical and structural aberrations observed (instability is represented by the first occurrence of a particular chromosomal alteration, assuming that the additional occurrences of the same alteration are in daughter cells.

**Supplementary Table S2** Chromosome instability in the four MEF genotypes (Set #1)


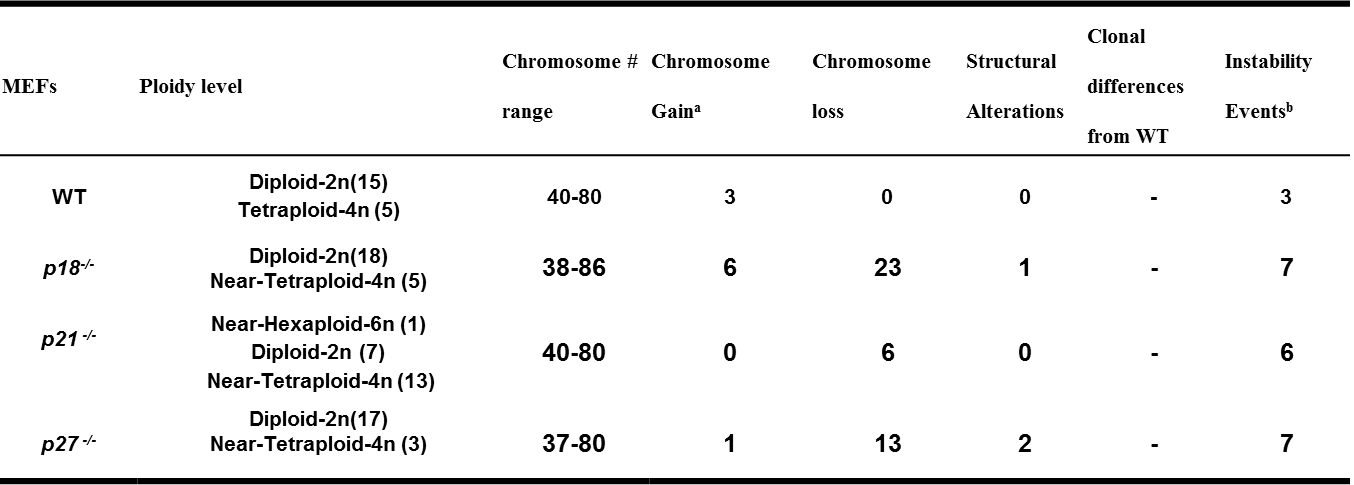


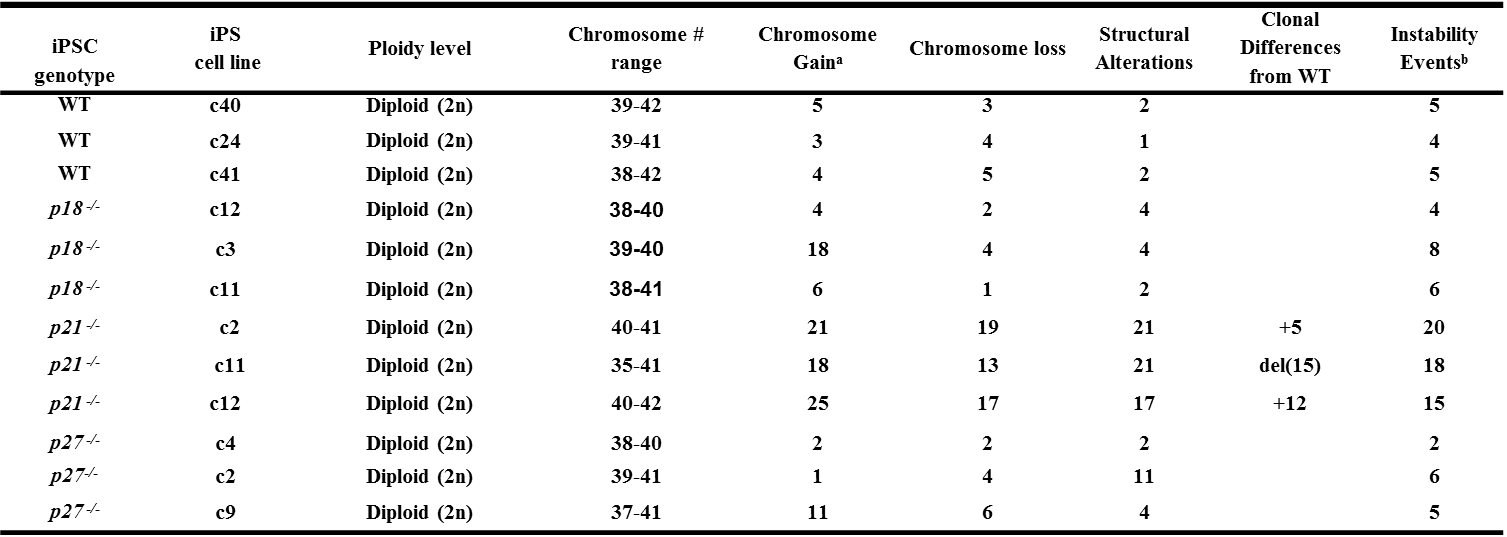
**Supplementary Table S3** Chromosome instability in the four iPSC genotypes (Set #2)

**Supplementary Table S4** Chromosome instability in the four MEF genotypes (Set #2)


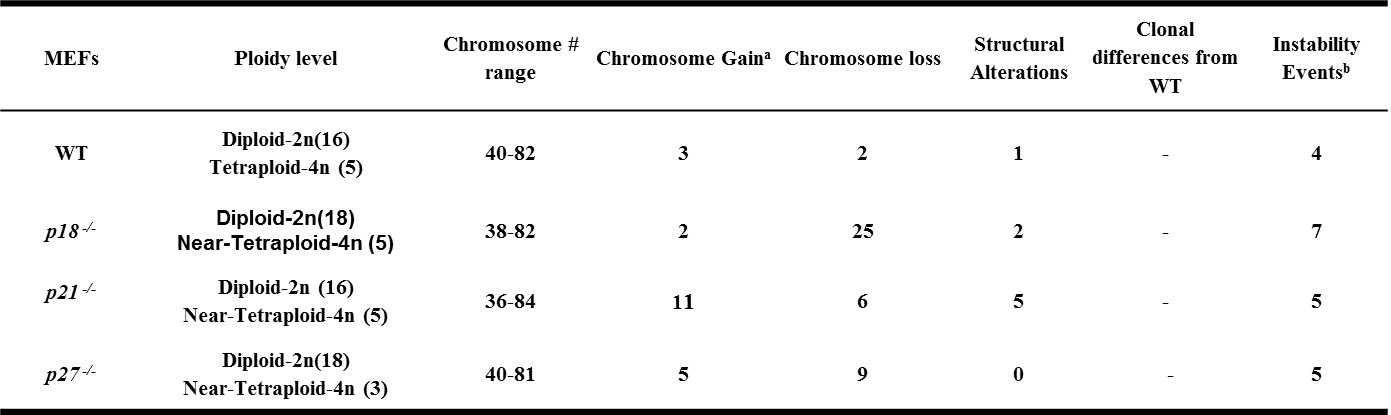


**Supplementary Table S5** Chromosome instability in the four iPSC genotypes (Set #3)


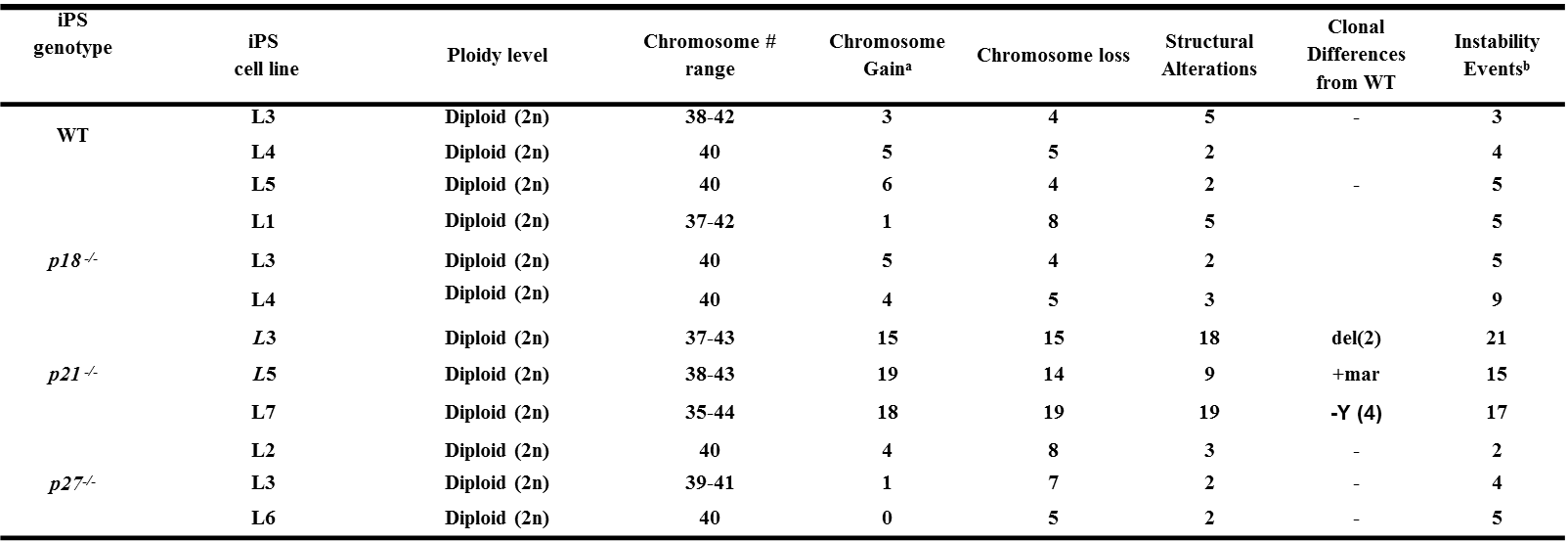


**Supplementary Table S6** Chromosome instability in the four MEF genotypes (Set #3)


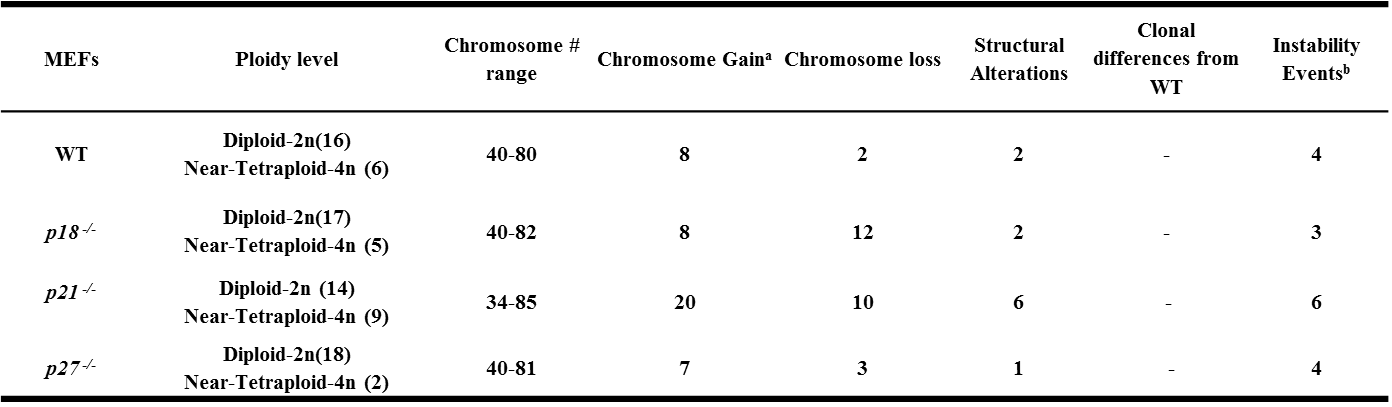

Supplement: Supplementary file 1 — Supplemental Tables 1-6 [file 41419_2019_1502_MOESM1_ESM.docx]
