# Supplementary material for: Absence of cyclin-dependent kinase inhibitor p27 or p18 increases efficiency of iPSC generation without induction of iPSC genomic instability
Source: Cell Death Dis. 2019 Mar 20;10(4):271. doi: 10.1038/s41419-019-1502-8 (PMC6426969; doi:10.1038/s41419-019-1502-8)
Supplement: Supplementary file 3 — Supplemental Figure 2 [file 41419_2019_1502_MOESM3_ESM.pdf]

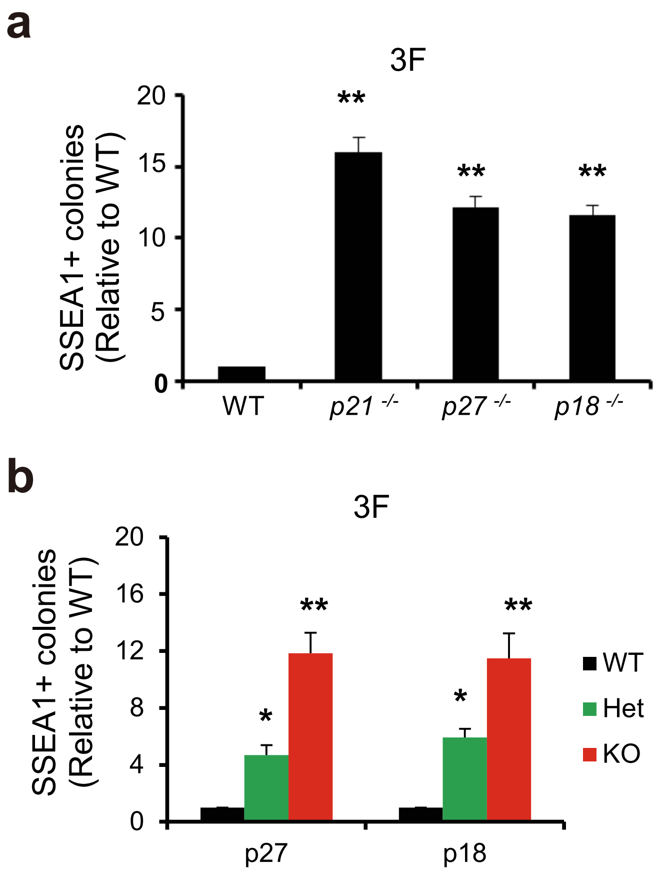

Supplemental Figure 2 Effects of absence of p21, p27, or p18 on iPSC generation by transduction with three factors (Oct4, Sox2 and Klf4).

a) SSEA1 positive colonies derived from different genotype MEFs relative to that from WT MEFs at reprogramming day 14 by transduction with three factors (Oct4, Sox2 and Klf4).

b) SSEA1 positive colonies in $p27^{+/-}$, $p27^{-/-}$, $p18^{+/-}$, and $p18^{-/-}$ iPSCs relative to that of WT iPSCs at day 14 after transduction with three factors.

Data are representative of two or three independent experiments. Error bars, ±SD. *, $p<0.05$; **, $p< 0.01$, by two-tailed *t* test.
