# Supplementary material for: Absence of cyclin-dependent kinase inhibitor p27 or p18 increases efficiency of iPSC generation without induction of iPSC genomic instability
Source: Cell Death Dis. 2019 Mar 20;10(4):271. doi: 10.1038/s41419-019-1502-8 (PMC6426969; doi:10.1038/s41419-019-1502-8)
Supplement: Supplementary file 4 — Supplemental Figure 3 [file 41419_2019_1502_MOESM4_ESM.pdf]

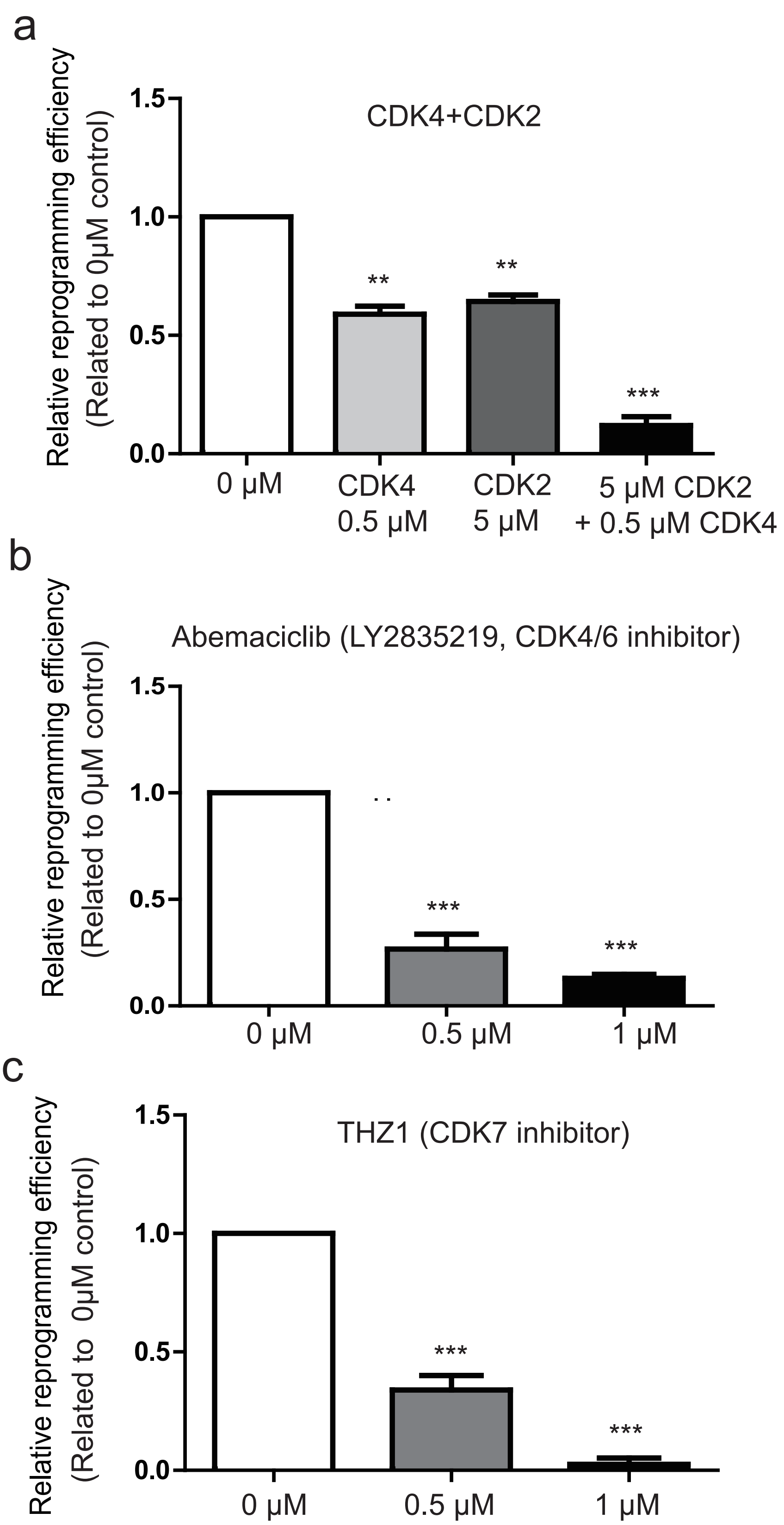

Supplemental Figure 3 Effects of CDK inhibitors on efficiency of iPSC generation in WT MEFs

a) Effects of CDK4 and/or CDK2 inhibitor treatment on efficiency of iPSC generation in WT MEFs.

b and c) Effects of CDK4/6 (b) and CDK7 (c) inhibitor treatment on efficiency of iPSC generation in WT MEFs. Inhibitors were added in the medium during reprogramming.

Data are representative of two or three independent experiments. Error bars, ±SD. **, *p*< 0.01, ***, *p*< 0.001by two-tailed *t* test.
